# Matrix Metalloproteinase-11 Promotes Early Mouse Mammary Gland Tumor Growth through Metabolic Reprogramming and Increased IGF1/AKT/FoxO1 Signaling Pathway, Enhanced ER Stress and Alteration in Mitochondrial UPR

**DOI:** 10.3390/cancers12092357

**Published:** 2020-08-20

**Authors:** Bing Tan, Amélie Jaulin, Caroline Bund, Hassiba Outilaft, Corinne Wendling, Marie-Pierrette Chenard, Fabien Alpy, A. Ercüment Cicek, Izzie J. Namer, Catherine Tomasetto, Nassim Dali-Youcef

**Affiliations:** 1 Institut de Génétique et de Biologie Moléculaire et Cellulaire Illkirch, 1 rue Laurent Fries, BP 10142, 67404 Illkirch CEDEX, France; tanbing3537@gmail.com (B.T.); jaulina@igbmc.fr (A.J.); wendling@igbmc.fr (C.W.); marie-pierrette.chenard@chru-strasbourg.fr (M.-P.C.); falpy@igbmc.fr (F.A.); 2 Centre National de la Recherche Scientifique, UMR 7104, 1 rue Laurent Fries, BP 10142, 67404 Illkirch CEDEX, France; 3 Institut National de la Santé et de la Recherche Médicale, U1258, 1 rue Laurent Fries, BP 10142, 67404 Illkirch CEDEX, France; 4 Université de Strasbourg, 1 rue Laurent Fries, BP 10142, 67404 Illkirch Cedex, France; 5 MNMS-Platform, Hôpital de Hautepierre, Hôpitaux Universitaires de Strasbourg, 1 avenue Molière, 67200 Strasbourg CEDEX, France; c.bund@icans.eu (C.B.); hassiba2a@live.fr (H.O.); ij.namer@icans.eu (I.J.N.); 6 Service de Médecine Nucléaire et d’Imagerie Moléculaire, Institut de Cancérologie Strasbourg Europe, 67200 Strasbourg, France; 7 ICube, Université de Strasbourg/CNRS, UMR 7357, 67091 Strasbourg Cedex, France; 8 Département de pathologie, Hôpitaux Universitaires de Strasbourg, Hôpital de Hautepierre, 1 avenue Molière, 67200 Strasbourg, France; 9 Computer Engineering Department, Bilkent University EA-514 Bilkent, Ankara 06800, Turkey; cicek@cs.bilkent.edu.tr; 10 Computational Biology Department, School of Computer Science, Carnegie Mellon University, 5000 Forbes avenue, Pittsburgh, PA 15213, USA; 11 Laboratoire de Biochimie et Biologie Moléculaire, Pôle de Biologie, Hôpitaux Universitaires de Strasbourg, Nouvel Hôpital Civil, 1 place de l’hôpital, 67091 Strasbourg CEDEX, France

**Keywords:** Warburg effect, breast cancer, UPR^ER^, UPR^mt^, metabolomics, metabolic flexibility

## Abstract

Matrix metalloproteinase 11 (MMP11) is an extracellular proteolytic enzyme belonging to the matrix metalloproteinase (MMP11) family. These proteases are involved in extracellular matrix (ECM) remodeling and activation of latent factors. MMP11 is a negative regulator of adipose tissue development and controls energy metabolism in vivo. In cancer, MMP11 expression is associated with poorer survival, and preclinical studies in mice showed that MMP11 accelerates tumor growth. How the metabolic role of MMP11 contributes to cancer development is poorly understood. To address this issue, we developed a series of preclinical mouse mammary gland tumor models by genetic engineering. Tumor growth was studied in mice either deficient (Loss of Function-LOF) or overexpressing MMP11 (Gain of Function-GOF) crossed with a transgenic model of breast cancer induced by the polyoma middle T antigen (PyMT) driven by the murine mammary tumor virus promoter (MMTV) (MMTV-PyMT). Both GOF and LOF models support roles for MMP11, favoring early tumor growth by increasing proliferation and reducing apoptosis. Of interest, MMP11 promotes Insulin-like Growth Factor-1 (IGF1)/protein kinase B (AKT)/Forkhead box protein O1 (FoxO1) signaling and is associated with a metabolic switch in the tumor, activation of the endoplasmic reticulum stress response, and an alteration in the mitochondrial unfolded protein response with decreased proteasome activity. In addition, high resonance magic angle spinning (HRMAS) metabolomics analysis of tumors from both models established a metabolic signature that favors tumorigenesis when MMP11 is overexpressed. These data support the idea that MMP11 contributes to an adaptive metabolic response, named metabolic flexibility, promoting cancer growth.

## 1. Introduction

Breast cancer is a leading cause of death by cancer in women [1]. In addition to cancer cells, the tumor microenvironment (TME) plays an important role in breast cancer progression [2]. The TME is composed of an extracellular matrix and distinct cell types including macrophages, immune cells, endothelial cells, and fibroblasts. Adipocytes are an emerging cellular component of the TME, they have a direct impact on cancer cells by cell-cell contacts, but also an indirect contribution by a paracrine action [3]. Matrix metalloproteinase-11 (MMP11), also called stromelysin-3, is a protein secreted by stromal cells during breast cancer invasion, and increased MMP11 levels have been associated with poor outcome in cancer patients [4,5,6,7]. In the context of breast cancer, MMP11 is a TME component expressed by cancer-associated fibroblasts (CAFs) in the tumor center and by cancer-associated adipocytes (CAAs) at the tumor invasive front [3,7,8]. In addition, MMP11 is expressed by breast cancer intratumoral mononuclear inflammatory cells and this expression is the most potent and independent factor to predict relapse-free survival and overall survival [9]. Despite its presence in the TME from many carcinomas, the contribution of MMP11 in this compartment remains elusive. We have previously shown that MMP11 acts as a negative regulator of adipogenesis both in vitro and in vivo [8,10,11]. More recently, we highlighted a physiological role of MMP11 as a metabolic regulator of whole-body energy homeostasis. Increased MMP11 expression (MMP11^Tg^) is associated with a lean phenotype and protection from diet-induced obesity, while loss of MMP11 expression (MMP11^KO^) promotes weight gain and metabolic syndrome [12]. This study showed that MMP11 mediates a metabolic switch to aerobic glycolysis at the expense of oxidative phosphorylation in the absence of cancer. In breast cancer, the metabolic role of MMP11 was not addressed and mechanisms behind MMP11-mediated breast tumor growth remain poorly comprehended. In order to tackle this issue, we used MMP11 gain-of-function (GOF, MMP11^Tg^) and loss-of-function (LOF, MMP11^KO^) mouse models to examine the role of MMP11 on mammary tumor growth and metabolism. We crossed these mice with a genetic model of spontaneous mammary tumors: the murine mammary tumor virus promoter (MMTV)-polyoma middle T antigen (PyMT) genetic strain [13,14]. MMTV-PyMT females develop with high penetrance palpable mammary tumors that metastasize to the lung [15]. In this study, we compare tumor growth in GOF and LOF for MMP11 expression. Both models were consistent and showed that MMP11 favors tumor growth at an early stage. Moreover, this study brings novel insight into the metabolic role of MMP11 in tumor growth through induction of metabolic reprogramming.

## 2. Results

### 2.1. MMP11 Decreases MMTV-PyMT Mice Body Weight and Increases Mammary Tumor Incidence

**Figure 1:**
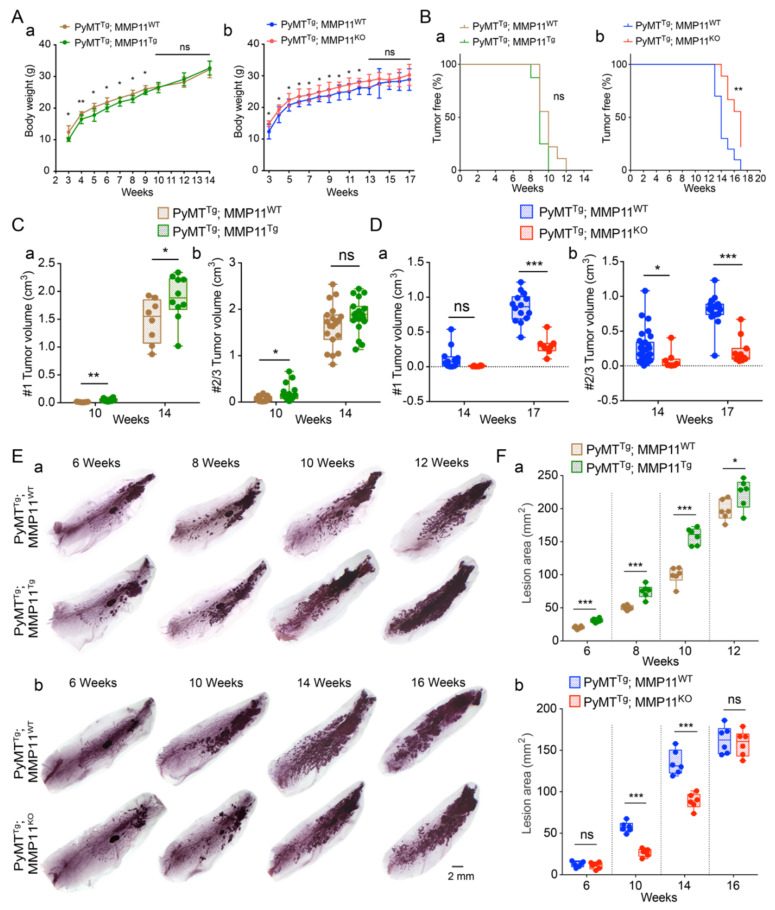
(**A**) Analysis of mice body weight in polyoma middle T antigen (PyMT)^Tg^; matrix metalloproteinase 11 (MMP11)^Tg^ (**a**) and PyMT^Tg^; MMP11^KO^ (**b**) animals as compared to their respective PyMT^Tg^ MMP11^WT^ controls. Overexpression of MMP11 decreased body weight before 9 weeks of age, whereas MMP11 inactivation increased mice body weight before 12 weeks of age; (**B**) Analysis of mice tumor development in 10- and 14-week-old PyMT^Tg^; MMP11^Tg^ (**a**) and in 14 and 17-week-old PyMT^Tg^; MMP11^KO^ (**b**) mice. (**a**) No difference of percentage of tumor-free mice between PyMT^Tg^; MMP11^Tg^ mice and controls; (**b**) PyMT^Tg^; MMP11^KO^ mice exhibited a significant increase in the percentage of tumor-free mice as compared to PyMTTg; MMP11^WT^ animals; (**C**,**D**) Analysis of tumor volume in mammary glands #1 (**a**) and #2/3 (**b**) in 10- and 14-week-old PyMT^Tg^; MMP11^Tg^ (**C**) and in 14- and 17-week-old PyMT^Tg^; MMP11^KO^ (**D**) mice, one dot corresponding to a palpable tumor; (**E**) Whole mount carmine-red staining of #4 mammary glands showed the developmental hyperplasia and neoplastic lesions at different time points in PyMT^Tg^; MMP11^Tg^ mice (**a**) and in PyMT^Tg^; MMP11^KO^ mice (**b**) as compared to their respective controls; (**F**) Quantification of the lesion area of hyperplasia and neoplasm as represented in E in PyMT^Tg^; MMP11^Tg^ mice (**a**) and in PyMT^Tg^; MMP11^KO^ mice (**b**) as compared to their controls. N = 6–8 mice/group, data are presented as mean ± standard error of the mean (SEM). * *p* < 0.05, ** *p* < 0.01, *** *p* < 0.001 (unpaired *t*-test).

To explore the role of MMP11 in cancer, we took advantage of two genetic models of gain and loss of function and crossed them with the mammary gland tumor prone MMTV-PyMT strain (Figure S1) [12,15]. In the MMP11^Tg^ model, MMP11 is expressed under the control of the keratin 14 promoter (Figure S1), this construct leads to the presence of the MMP11 protein in the skin, a highly vascularized tissue (Figures S1, S2A, and S2B), and results in a complete body exposure to MMP11 [12]. Of note, the size of the MMP11 protein detected in the skin of MMP11^Tg^ indicates that the protein is predominantly present under its pro-form (Figure S2B). The MMP11^KO^ model is a constitutive knock-out resulting in the complete loss of MMP11 expression [12] (Figure S1). In a non-cancer model, we showed that MMP11^Tg^ mice are leaner than their control counterparts and reciprocally that MMP11^KO^ animals are overweight [12]. We examined body weight in double transgenic mice either expressing or lacking MMP11 in the presence of the Py-MT transgene (Figure 1). In the GOF breast cancer model PyMT^Tg^; MMP11^Tg^, MMP11 decreased postnatal body weight of MMTV-PyMT mice from 3 to 9 weeks of age but no difference was noticed after palpable tumor occurred at 9–10 weeks of age (Figure 1Aa). In the LOF model (PyMT^Tg^; MMP11^KO^), MMP11 inactivation was accompanied by increased postnatal mice body weight from 3 to 12 weeks of age but no difference was observed after palpable tumor occurred at 13–14 weeks of age (Figure 1Ab). The changes in body weight observed in the GOF and LOF breast cancer models recapitulated those already reported in single MMP11-overexpressed and -inactivated mice models [12]. Even though the tumor grew rapidly to a certain extent in PyMT^Tg^; MMP11^Tg^ mice, tumor incidence was not significantly different from PyMT^Tg^; MMP11^WT^ control mice (Figure 1Ba). However, tumor growth was significantly delayed in PyMT^Tg^; MMP11^KO^ as compared to PyMT^Tg^; MMP11^WT^ controls (Figure 1Bb).

Our results show that MMP11 affects body weight and tumor incidence in MMTV-PyMT mice at an early stage of tumor development.

### 2.2. MMP11 Promotes MMTV-PyMT Mice Mammary Tumor Growth

To assess the impact of MMP11 on tumor development, we measured palpable tumor sizes with a caliper in individual mammary glands of PyMT^Tg^; MMP11^Tg^ and PyMT^Tg^; MMP11^KO^ mice and compared them with their respective controls because both lines are on a different genetic background (Figure S1Bb’,Bb). Mice have ten mammary glands organized in pairs. Tumor development is not synchronous among the different pairs and studies showed that in the PyMT model the distinct mammary gland pairs have different tumor-initiating properties [16]. Thus, we evaluated tumor growth in individual mammary glands, except for mammary glands #2 from #3, which are indistinguishable. Therefore, they were considered as one entity #2/3. Overexpression of MMP11 increased tumor volume in #1, #2/3, and #4 mammary glands in 10-week-old PyMT^Tg^; MMP11^Tg^ mice, and in #1 mammary gland in 14-week-old animals as compared to controls (Figure 1Ca,Cb and Figure S2C). Conversely, in PyMT^Tg^; MMP11^KO^ mice, we observed a decrease in tumor volume in #2/3 and #4 mammary glands in 14-week-old mice, and in #1, #2/3 mammary glands in 17-week-old animals as compared to controls (Figure 1Da,Db and Figure S2C). Then, we examined the tumor lesions and performed whole mount carmine-red staining in the #4 mammary gland to assess hyperplastic and neoplastic lesion areas both in GOF and LOF double transgenic mice at different time points. We chose mammary gland #4 because the fat pad is well developed (Figure S1C). Quantification of carmine red staining indicated that PyMT^Tg^; MMP11^Tg^ mice had increased lesion area at 6, 8, 10, and 12 weeks of age (Figure 1Ea,Fa for quantification), whereas PyMT^Tg^; MMP11^KO^ mice had smaller lesions at 10 and 14 weeks of age as compared to their respective control littermates (Figure 1Eb,Fb for quantification). In both models, the tumor burden was similar at later stages close to the experimental end-point.

Together, these data demonstrate that MMP11 accelerates MMTV-PyMT transgenic mice tumor development and growth at early stage.

### 2.3. MMP11 Reduces Necrosis and Apoptosis in Early Stage Mammary Gland Tumor Development in MMTV-PyMT Mice

**Figure 2:**
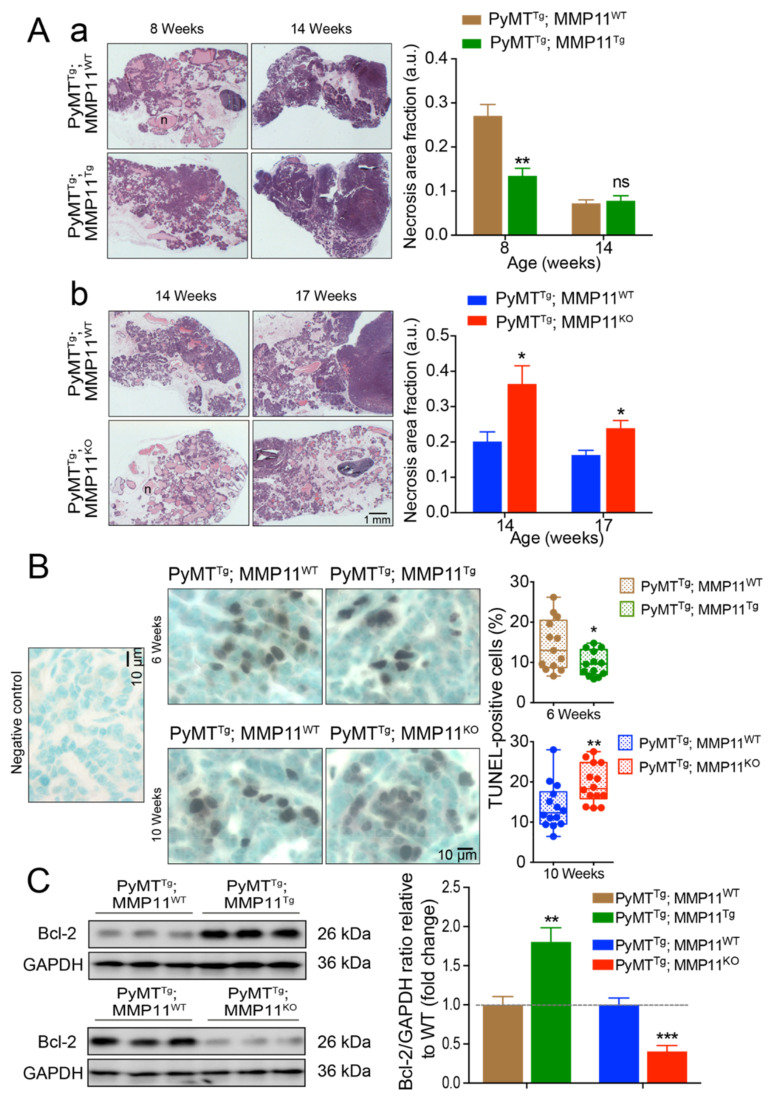
MMP11 plays anti-necrosis and anti-apoptotic roles in early stage of tumor development. (**A**) (**a**) Hematoxylin and eosin (HE) staining shows decreased tumor necrosis area in 8-week-old PyMT^Tg^; MMP11^Tg^ mice but no change in necrosis area at 14-week-old mice as compared to in PyMT^Tg^; MMP11^WT^ age-matched mice; (**b**) 14- and 17-week-old PyMT^Tg^; MMP11^KO^ mice exhibit increased necrosis area as compared to control mice; (**B**) Apoptosis was measured by the TUNEL-assay apoptotic cells in tumors from 6-week-old PyMT^Tg^; MMP11^Tg^ mice as compared to age-matched controls (upper panel), whereas 10-week-old PyMT^Tg^; MMP11^KO^ mice exhibit a significant increase in apoptotic cell percentage as compared to control animals (lower panel); (**C**) Immunoblots of tumor protein extracts show an increase in the expression of the anti-apoptotic protein Bcl-2 in 6-week-old PyMT^Tg^; MMP11^Tg^ mice as compared to controls, while a decrease in Bcl-2 is observed in tumor protein extracts from PyMT^Tg^; MMP11^KO^ mice as compared to controls (left panel). Quantification of immunoblot signal density normalized to glyceraldehyde-3-phosphate dehydrogenase (GAPDH) protein levels relative to wild type is depicted in the right panel. N = 6–8 mice/group, data are expressed as mean ± SEM. * *p* < 0.05, ** *p* < 0.01, *** *p* < 0.001 (unpaired *t*-test).

To determine the impact of MMP11 on tumor necrosis and apoptosis in MMTV-PyMT mice, we analyzed paraffin-embedded #4 mammary gland tissue sections from PyMT^Tg^; MMP11^Tg^ and PyMT^Tg^; MMP11^KO^ mice and their control littermates by hematoxylin and eosin (HE) staining and Terminal deoxynucleotidyl transferase dUTP Nick End Labeling (TUNEL) assay. Using HE staining, cell death and cytolysis (necrosis) are visualized by the light pink color while viable tumor cells are darker (purple color). PyMT^Tg^; MMP11^Tg^ mice displayed decreased necrosis area at 8 weeks of age, but no significant difference in necrosis was observed at a later stage (14 weeks) as compared to PyMT^Tg^; MMP11^WT^ mice (Figure 2Aa). Consistently, PyMT^Tg^; MMP11^KO^ mice exhibited an increase in necrosis area at 14 and 17 weeks of age as compared to controls (Figure 2Ab). We then quantified the ratio of apoptotic cells in GOF and LOF mice as compared to their respective controls by TUNEL assay. We observed a significant decrease in the percentage of apoptotic cells in PyMT^Tg^; MMP11^Tg^ mice as compared to PyMT^Tg^; MMP11^WT^ control mice at 6 weeks of age but not at a later stage (10 weeks), whereas PyMT^Tg^; MMP11^KO^ mice displayed a significant increase in the proportion of TUNEL positive cells at 10 weeks of age (Figure 2B). Invariably, the expression level of the anti-apoptotic protein Bcl-2 was increased in PyMT^Tg^; MMP11^Tg^ mice and decreased in PyMT^Tg^; MMP11^KO^ mice as compared with their respective controls (Figure 2C and Figure S3).

To conclude, at early stage of mammary gland tumor development, the presence of MMP11 decreases cell death and hence confers a survival advantage to tumors, thereby supporting cancer growth.

### 2.4. MMP11 Promotes Cell Proliferation in MMTV-PyMT Mice Mammary Tumor Development at an Early Stage and Induces the Insulin-Like Growth Factor-1 Signaling Pathway

**Figure 3:**
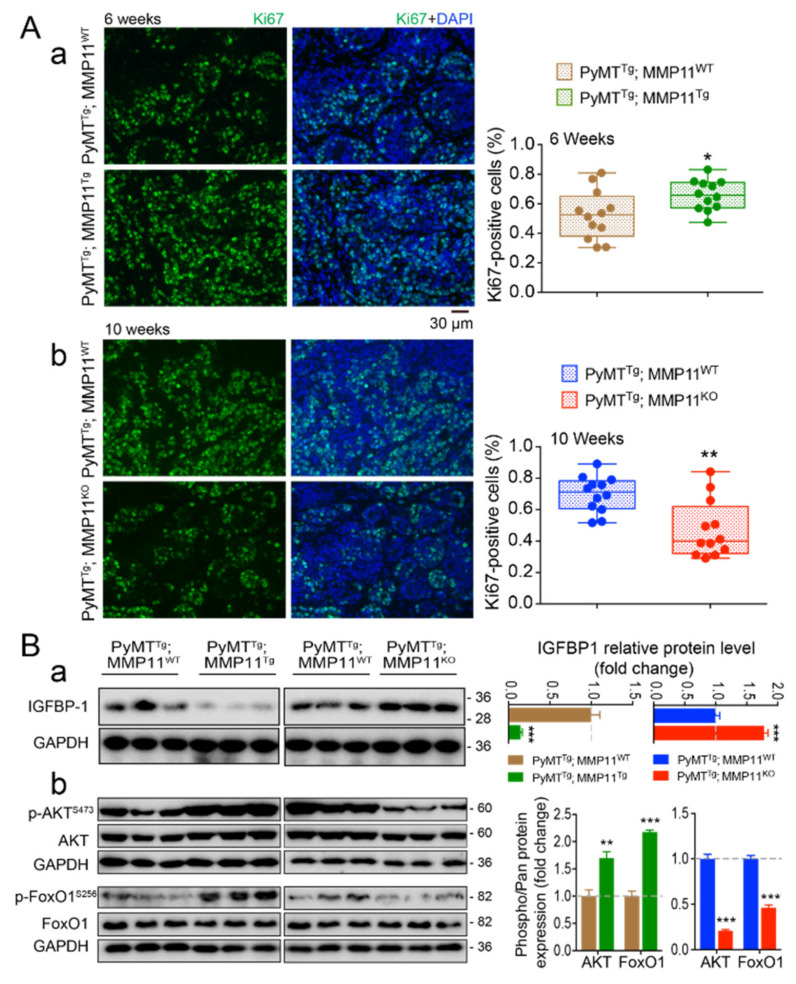
(**A**) MMP11 promotes tumor cell proliferation at early stage of tumor development. Immunofluorescence staining shows increased percentage of Ki-67-positive cells in the tumors of 6-week-old PyMT^Tg^; MMP11^Tg^ mice (**a**), whereas tumors from 10-week-old PyMT^Tg^; MMP11^KO^ animals (**b**) have less proliferating cells compared with their respective controls; (**B**) Left panel: Western blot protein expression profiles of Insulin-like growth factor-binding protein 1 (IGFBP1) (**a**) and of components of its downstream IGF1/AKT/FoxO1 signaling pathway (**b**) reveal activation of this cascade in representative tumors from 6-week-old PyMT^Tg^; MMP11^Tg^ mice and its decrease in tumors from 10-week-old PyMT^Tg^; MMP11^KO^ animals as compared to their respective control animals. Right panel: quantification of the ratios of phosphorylated proteins to total level compared to wildtype are presented, n = 6–8 mice/group, data are expressed as mean ± SEM, * *p* < 0.05, ** *p* < 0.01, *** *p* < 0.001 (unpaired *t*-test).

To investigate the impact of MMP11 on tumor proliferating cells, tumor tissue sections were analyzed by immunofluorescence using an antibody against the cell proliferation marker Ki-67, a protein encoded by the MKI67 gene [17]. The proportion of Ki-67-positive cells was significantly increased in PyMT^Tg^; MMP11^Tg^ as compared to PyMT^Tg^; MMP11^WT^ mice in 6-week-old mice (Figure 3Aa), whereas PyMT^Tg^; MMP11^KO^ mice had less Ki-67-positive cells compared to their wildtype controls at 10 weeks of age (Figure 3Ab). However, at a later stage (8 weeks of age), PyMT^Tg^; MMP11^Tg^ tumors had a similar percentage of proliferating cells as compared to PyMT^Tg^; MMP11^WT^ tumors (data not shown). These data show that MMP11 increases PyMT tumor cell proliferation at an early stage of tumor development.

Given that the IGF1 pathway is a recognized stimulator of cell growth and plays an important role in neoplasia [18], and that IGF1 bioavailability is increased upon MMP11 overexpression [12], we investigated whether the IGF1 signaling pathway is exacerbated in tumor extracts from 6-week-old PyMT^Tg^; MMP11^Tg^ mice. Two important components of this cascade, namely protein kinase B (AKT) and its target Forkhead box protein O1 (FoxO1), were more phosphorylated in tumor protein extracts from PyMT^Tg^; MMP11^Tg^ mice as compared to control mice (Figure 3Bb, left blot and Figure S4). Consistently, phosphorylation of AKT and FoxO1 were decreased in PyMT^Tg^; MMP11^KO^ mice compared to controls (Figure 3Bb, right blot and Figure S4). Given that Insulin-like growth factor-binding protein 1 (IGFBP1) is a known substrate of MMP11 [19], it is likely that MMP11 activates the IGF1/AKT growth pathway by increasing IGF1 bioavailability. In agreement with this hypothesis, the abundance of IGFBP1, the protein that controls the circulating levels of IGF1 [20], is reduced in PyMT^Tg^; MMP11^Tg^ tumors (Figure 3Ba, left blot) and increased in PyMT^Tg^; MMP11^KO^ tumors (Figure 3Ba, right blot and Figure S4).

Collectively, these results reveal that MMP11 overexpression promotes cell proliferation in PyMT^Tg^; MMP11^Tg^ tumors and exacerbates the IGF1/AKT/FoxO1 pathway. They support the notion that in the presence of MMP11, IGF1 increases cell proliferation.

### 2.5. MMP11 Increases Lipid Uptake and Utilization and Promotes Metabolic Reprogramming

**Figure 4:**
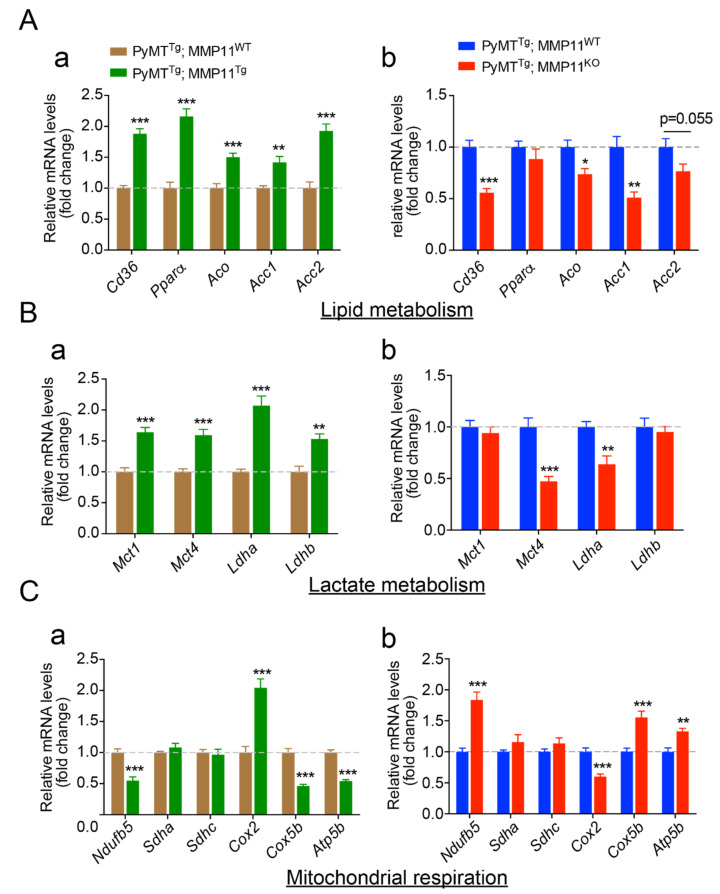
MMP11 promotes mammary gland tumor metabolic switch and lipid utilization. RT-qPCR transcripts of genes involved in (**A**) lipid metabolism, (**B**) lactate metabolism, and (**C**) mitochondrial respiration in tumor extracts from both gain-of-function (GOF) (PyMT^Tg^; MMP11^Tg^, (**a**) left panels) mice and loss-of-function (LOF) (PyMT^Tg^; MMP11^KO^, (**b**) right panels) mice models. Data are presented as fold changes to control. N = 6–8 mice/group, data are presented as mean ± SEM, * *p* < 0.05, ** *p* < 0.01, *** *p* < 0.001 (unpaired *t*-test).

Cancer cells exhibit a reprogrammed cell metabolism compared to normal cells resulting from specific enhanced energy demands [21]. In the light of the physiological role of MMP11 [12], we hypothesized that MMP11 stimulates lipid utilization and promotes aerobic glycolysis, a process known as the Warburg effect, to support tumor growth [21]. To address the metabolic role of MMP11 in tumors, we measured the expression of key metabolic genes in tumor samples. Regarding lipid metabolism, in tumor samples from 6-week-old PyMT^Tg^; MMP11^Tg^ mice, we observed a significant increase in the expression of the fatty acid transporter *Cd36* and the oxidative genes peroxisome proliferator-activated receptor alpha (*Pparα*) and its target gene acyl coenzyme A oxidase (*Aco*), but also increased expression of acetyl-Coenzyme A carboxylase 1 and 2 (*Acc1* and *Acc2*), supporting an increased lipid uptake, utilization, and turnover (Figure 4Aa) [22,23,24]. Consistently, we observed the mirror expression profile in tumors from 10-week-old PyMT^Tg^; MMP11^KO^ mice as compared to their control littermates, with the exception that *Pparα* expression was not altered in this case (Figure 4Ab). Next, because in cancer cells aerobic glycolysis (Warburg effect) becomes the major metabolic pathway generating lactate at the expense of oxidative phosphorylation [21], we analyzed the expression of genes implicated in lactate metabolism and observed an increase in the expression of genes involved in lactate production: lactate dehydrogenase A (*Ldha*), release: monocarboxylate transporter 4 (*Mct4*), uptake: monocarboxylate transporter 1 (*Mct1*), and metabolism: lactate dehydrogenase B (*Ldhb*) in PyMT^Tg^; MMP11^Tg^ mice as compared to controls (Figure 4Ba) [25,26]. Interestingly, a significant reduction in the expression of *Mct4* and *Ldha* was found in PyMT^Tg^; MMP11^KO^ tumors, suggesting a reduction in lactate production and release. No significant difference was observed in the expression of *Mct1* and *Ldhb* (Figure 4Bb). To determine whether the increase in aerobic glycolysis seen in PyMT^Tg^; MMP11^Tg^ mice is accompanied by negative regulation of certain oxidative phosphorylation (OXPHOS) genes consistent with Warburg’s concept, we analyzed the expression of genes involved in the mitochondrial electron transport chain (ETC). In PyMT^Tg^; MMP11^Tg^ tumors a significant decrease in the expression of *Ndufb5*, a gene that encodes a subunit of complex I of the mitochondrial respiratory chain, which transfers electrons from NADH to ubiquinone; a decrease in *Cox5b* expression, a gene encoding the cytochrome c subunit 5b protein of complex IV of the ETC; and a diminished expression of *Atp5b*, a gene encoding ATP synthase subunit 5 b of complex V were observed (Figure 4Ca). The alteration of these genes suggests that in the presence of MMP11, tumor cells have a decrease in mitochondrial respiration. A mirror phenotype was found in tumors from PyMT^Tg^; MMP11^KO^ mice, with increased *Ndufb5*, *Cox5b*, and *Atp5b* expression (Figure 4Cb). Of note, as compared to controls, tumors from PyMT^Tg^; MMP11^Tg^ mice had increased expression of the mitochondrial encoded gene *Cox2* (cytochrome c oxidase subunit 2, complex IV), suggesting an increase in mitochondria number (Figure 4Ca). Reciprocally, the expression of *Cox2* was diminished in PyMT^Tg^; MMP11^KO^ tumors compared to control specimen (Figure 4Cb).

Taken together, these data suggest that MMP11 overexpression confers an advantage for cancer cells to promote their growth through a metabolic reprogramming involving an increase in aerobic glycolysis, a decrease in mitochondrial respiration, and an increase in lipid turnover.

### 2.6. MMP11 Increases Endoplasmic Reticulum Stress Response and Alters Mitochondrial Unfolded Protein Response (UPR^mt^)

**Figure 5:**
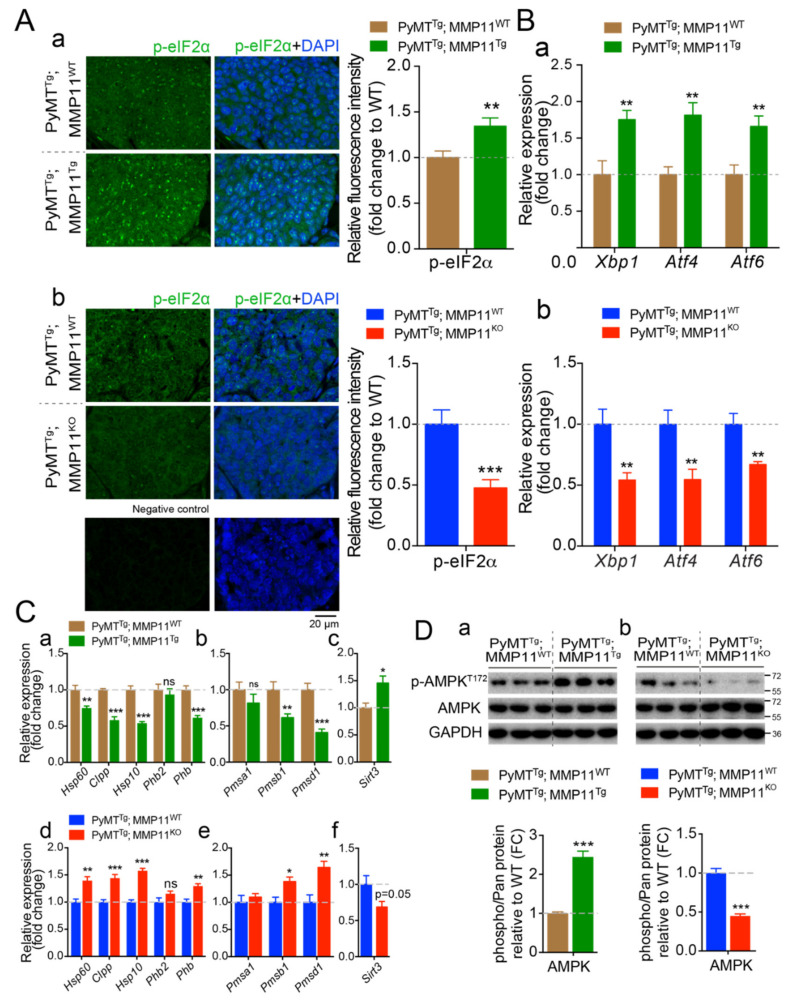
MMP11 promotes tumor cellular endoplasmic reticulum (ER) stress response (UPR^ER^) and alters mitochondrial UPR (UPR^mt^) in mammary gland tumors. (**A**) (**a**) Confocal images of tumor sections were stained using an anti-phosphorylated α subunit of eukaryotic translation initiation factor 2 (eIF2α) (*p*-eIF2α) antibody, from 12-week-old PyMT^Tg^; MMP11^Tg^ and control age-matched mice; (**b**) Confocal images of tumor sections stained with anti-*p*-eIF2α from 14-week-old PyMT^Tg^; MMP11^KO^ and control; a control staining without primary antibody is shown below; (**a**,**b**) relative image quantification are presented as histograms in the right; (**B**) (**a**) RT-qPCR analysis of genes implicated in UPR^ER^ in tumors from 6-week-old PyMT^Tg^; MMP11^Tg^ mice compared to tumors from control mice; (**b**) and in tumors from PyMT^Tg^; MMP11^KO^ mice compared to controls; (**C**) (**a**–**c**) Expression profile of genes implicated in the three arms of the UPR^mt^ in tumor samples from 6-week-old PyMT^Tg^; MMP11^Tg^ mice compared to controls; (**d**–**f**) Expression profile of genes implicated in the three arms of the UPR^mt^ in tumor samples from 10-week-old PyMT^Tg^; MMP11^KO^ mice compared to controls; (**D**) Western blot analysis of phosphorylated AMP-activated kinase (AMPK) (pAMPK^T172^) in tumors from (**a**) 6-week-old PyMT^Tg^; MMP11^Tg^ and (**b**) 10-week-old PyMT^Tg^; MMP11^KO^ mice, respectively, as compared to their controls. Quantification of the ratios of pAMPK/AMPK is presented below the blots, normalized to GAPDH expression. Data are presented as fold changes. N = 6–8 mice/group, data are presented as mean ± SEM, * *p* < 0.05, ** *p* < 0.01, *** *p* < 0.001 (unpaired *t*-test).

Protein homeostasis or proteostasis is supported by a coordinated regulation of polypeptide production, folding, trafficking, and degradation when unfolded or misfolded proteins accumulate within the cell. The endoplasmic reticulum (ER) ensures proper folding and processing of proteins that will be secreted and hence is a guarantor of proteostasis [27,28]. In immune and metabolic cells, but also following the exposure of cells to a variety of stressors, proteostasis is disrupted and an ER unfolded protein response (UPR^ER^) is triggered off to restore protein homeostasis [28,29]. In our study, we studied the impact of MMP11 on ER stress and the UPR^ER^ in tumor samples from 6-week-old PyMT^Tg^; MMP11^Tg^ and 10-week-old PyMT^Tg^; MMP11^KO^ mice and their respective controls. Interestingly, PyMT^Tg^; MMP11^Tg^ tumors have an increase in the phosphorylation of the α subunit of eukaryotic translation initiation factor 2 (eIF2α), a target of the protein kinase RNA (PKR)-like ER kinase (PERK), and an important sensor of ER stress [28] (Figure 5Aa). Expression of UPR^ER^ driver genes such as X-box binding protein (*Xbp1*), activating transcription factor 4 (*Atf4*), and activating transcription factor 6 (*Atf6*) were increased in tumors from PyMT^Tg^; MMP11^Tg^ mice as compared to PyMT^Tg^; MMP11^WT^ controls (Figure 5Ba). Conversely, eIF2α phosphorylation (Figure 5Ab) and expression of *Xbp1*, *Atf4*, and *Atf6* were decreased in PyMT^Tg^; MMP11^KO^ tumors as compared to controls (Figure 5Bb).

Similarly to the ER, the mitochondrion is an organelle that possesses a unique protein folding quality control, named the mitochondrial unfolded protein response or UPR^mt^, that in response to stress ensures, through mitochondrial-nuclear communication, proper folding of imported proteins from the cytosol [30]. Indeed, when misfolded proteins accumulate in mitochondria or in case of dysfunctional mitochondria, the UPR^mt^ is activated. Previous studies showed that in cancer, the UPR^mt^ could play an important role to adapt to stress and maintain mitochondria integrity when oxidative stress rises (reviewed in Reference [31]). The three main branches/axes of the UPR^mt^ are the CCAAT-enhancer-binding protein homologous protein (CHOP)/Heat Shock Protein (HSP)/protease caseinolytic mitochondrial matrix peptidase proteolytic subunit (ClpP), the ERα/NRF1/proteasome and the SIRT3/FoxO3a/superoxide dismutase 2 (SOD2) arms [31]. Here, an alteration of the CHOP/HSP/ClpP pathway was found in tumors from 6-week-old PyMT^Tg^; MMP11^Tg^ mice as compared to PyMT^Tg^; MMP11^WT^ age-matched animals. Hence, expression levels of the genes in this pathway such as mitochondrial heat shock protein genes *Hsp60* and *Hsp10* were significantly decreased as well as those of the mitochondrial protease caseinolytic mitochondrial matrix peptidase proteolytic subunit (*Clpp*) and of the prohibitin (*Phb*), a sensor of mitochondria stress and a promoter of longevity [32] (Figure 5Ca). Since misfolded proteins are targeted for degradation by the ubiquitin-proteasome system (UPS), we studied whether the abnormal UPR^mt^ is accompanied by a normal elimination of misfolded proteins. Interestingly, MMP11 overexpression in the PyMT cancer model led to an abnormal proteasomal activity as shown by decreased expression of genes encoding the proteasomal subunits *Psmb1* and *Psmd1* in 6-week-old PyMT^Tg^; MMP11^Tg^ mice as compared to control animals (Figure 5Cb), suggesting that an accumulation of misfolded proteins could be responsible for the impaired mitochondrial function. Given that transcripts of the CHOP and proteasome arms were decreased, we analyzed the expression of *Sirt3*, a gene encoding a NAD^+^-dependent deacetylase, representing the third arm of the UPR^mt^. Interestingly, we noted an increase in the expression of *Sirt3* in tumors from 6-week-old PyMT^Tg^; MMP11^Tg^ mice as compared to controls (Figure 5Cc). Consistently, the opposite phenomenon was observed in tumors from 10-week-old PyMT^Tg^; MMP11^KO^ mice. They had an increased UPR^mt^ response with enhanced expression of *Hsp60*, *Hsp10,* and *Clpp* (Figure 5Cd), enhanced proteasomal activity with increased expression of *Psmb1* and *Psmd1* (Figure 5Ce), and decreased expression of *Sirt3* in comparison to control tumors from PyMT^Tg^; MMP11^WT^ animals (Figure 5Cf).

Previous reports demonstrated that mitochondrial dysfunction induced by electron transport chain (ETC) perturbations and reactive oxygen species (ROS) generation inhibits proteasomal activity through proteasome disassembly in different organisms including mammalian cells [33,34]. Since alterations in ETC leads to decreased ATP production [35] and that the UPS relies on ATP for its function, we tested whether MMP11-induced AMP-activated kinase (AMPK) activation was implicated in this process. Interestingly, we observed a significant increase in AMPK phosphorylation at threonine 172 residue in tumors from 6-week-old PyMT^Tg^; MMP11^Tg^ mice as compared to their controls (Figure 5Da and Figure S5). Consistently, tumors from 10-week-old PyMT^Tg^; MMP11^KO^ mice had a significant decrease in AMPK activity compared to control animals (Figure 5Db and Figure S5).

Overall, MMP11 exacerbates endoplasmic reticulum stress, alters the mitochondrial UPR, and impairs proteasome activity that might be caused by increased oxidative stress, contributing thereby in energy depletion and activation of AMPK. Reduction of AMPK activity agrees with the tumor phenotype and the high energy demand necessary for tumor growth.

### 2.7. MMP11 Induces a Change in Tumor Metabolomic Profile in Mice

**Figure 6:**
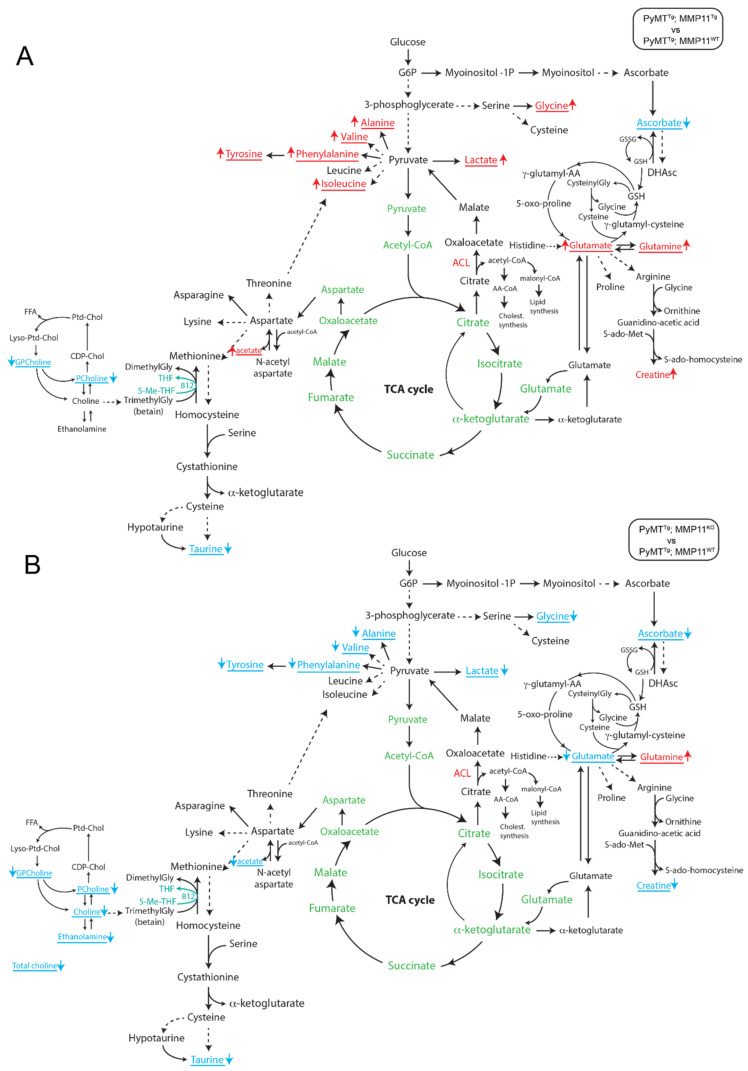
Results of the algorithm to determine the expected metabolite level alterations (ADEMA) network analysis of NMR high resonance magic angle spinning (HRMAS) metabolomics profile of mammary gland tumors in mice: (**A**) between PyMT^Tg^; MMP11^Tg^ mice compared to PyMT^Tg^; MMP11^WT^ controls and (**B**) between PyMT^Tg^; MMP11^KO^ compared to PyMT^Tg^; MMP11^WT^ controls. The metabolites underlined and written with an arrow for each one indicate the metabolites that are predicted to increase and decrease in PyMT^Tg^; MMP11^Tg^ (**A**) or PyMT^Tg^; MMP11^KO^ (**B**). The metabolites in green are present in the mitochondria. GSH: reduced glutathione; GSSG: oxidized glutathione; DHAsc: dehydroascorbate; g-glutamyl-AA: g-glutamyl-amino acid; Gly: glycine; ACL: ATP citrate lyase; AA-CoA: acetoacetyl-CoA.

To study the impact of MMP11 on tumor metabolism, we used high resonance magic angle spinning (HRMAS) nuclear magnetic resonance (NMR) spectroscopy to assess the metabolomic profile of tumor samples from 6-week-old PyMT^Tg^; MMP11^Tg^ and 10-week-old PyMT^Tg^; MMP11^KO^ mice as compared to their respective controls. Network analysis of the metabolomic profile of tumors using the algorithm to determine the expected metabolite level alterations (ADEMA) showed differences between PyMT^Tg^; MMP11^Tg^ and PyMT^Tg^; MMP11^KO^ mice compared with their respective controls. ADEMA allows the evaluation of changes in the concentration of an ensemble of metabolites between two experimental groups instead of analyzing the concentration of individual metabolites [36]. This analysis revealed that MMP11 overexpression in PyMT^Tg^; MMP11^Tg^ tumor samples is associated with a significant predicted decrease in ascorbate, glycerophosphocholine (GPcholine), phosphocholine (PCholine), and taurine with a predicted increase in lactate, glycine, glutamate, glutamine, creatine, and the amino acids alanine, valine, phenylalanine, tyrosine, and isoleucine as well as acetate as compared to control samples (Figure 6A). MMP11 deficiency in PyMT^Tg^; MMP11^KO^ tumors was accompanied by a predicted decrease in GPcholine, Pcholine, choline, ethanolamine, taurine, ascorbate, and acetate as well as lactate, creatine, glycine, alanine, valine, phenylalanine, tyrosine, and glutamate, while glutamine is predicted to increase in comparison to tumors from control animals (Figure 6B).

The changes observed in the metabolomic profile of tumors from PyMT^Tg^; MMP11^Tg^ mice evoke aggressiveness. Notably, lactate, a well-recognized feature of cancer cells as described by Warburg’s work, is predicted to increase, so is glutamate suggestive of increased glutaminolysis, a feature of highly proliferative cancer cells [21]. In PyMT^Tg^; MMP11^KO^ mice, we observed a reciprocal phenomenon with a prediction to decrease in lactate levels suggestive of mitigation of aerobic glycolysis when MMP11 is absent. Another example is the MMP11-mediated prediction to increase in glycine, a metabolite produced from serine by a branch of aerobic glycolysis, which provides carbon units for one-carbon metabolism. Amplification of this pathway has been associated with increased tumorigenesis in breast cancer [37,38,39].

Altogether, these results show that the presence of MMP11 is associated with a metabolomic signature favoring tumor growth.

## 3. Discussion

We showed previously that MMP11 functions in the regulation of whole-body metabolism through activation of the IGF1/AKT/FoxO1 signaling cascade in a non-cancer context using gain- and loss-of-function genetic-engineered mice models [12]. In this study, we show that in the MMTV-PyMT model, early mammary gland tumor incidence and growth are accelerated and delayed in the presence and in the absence of MMP11, respectively. Given that MMP11 is implicated in normal post-natal development of the mammary gland through a paracrine mechanism [40] the observed phenotypes may be, at least in part, linked to this developmental function. Nevertheless, the present study brings new insights into the role of MMP11 during cancer development in the spontaneous genetic MMTV-PyMT breast cancer mouse model. Hence, we report that in the presence of MMP11, the IGF1/AKT signaling pathway is activated, that is achieved through decreased IGFBP1 protein levels. MMP11 promotes tumor cell survival and significantly increases cell proliferation in tumor samples from PyMT^Tg^; MMP11^Tg^ mice as compared to control PyMT^Tg^; MMP11^WT^ animals, thereby accelerating tumor growth. Reciprocally, MMP11 deficiency results in a mirror phenotype and delayed tumor growth. In the presence of MMP11, the Warburg-like effect is increased in tumor samples through a metabolic reprogramming. Oxidative phosphorylation and aerobic glycolysis are reduced and increased, respectively, most likely through the IGF1/AKT/FoxO1 cascade. Besides lactate utilization, in the presence of MMP11 lipid turnover is increased (e.g., synthesis, transport, and metabolism) to serve as an additional nutrient source to fulfill tumor energetic needs allowing new membrane formation and expansion [41]. Interestingly, the presence of MMP11 correlates with ER stress through the significant elevation in the expression of *Atf4*, *Atf6,* and *Xbp1*, key components of the UPR^ER^ [28]. The increase in the phosphorylation of eIF2α, an upstream inducer of ATF4 and downstream effector of the ER stress-mediated kinase PERK [28], provides an additional evidence about the role of MMP11 in UPR^ER^. There is compelling evidence that UPR^ER^ is involved in tumorigenesis, more specifically in HER2-positive breast cancer [42], and hence constitutes an attractive target for anticancer treatment [43]. Likewise, the hypoxic environment of the tumors has a dual supportive role in cancer. On one hand, it stimulates the inositol requiring enzyme 1α (IRE1) and PERK branches of the UPR^ER^ to enhance the insensitivity of cancer cells to apoptosis [44]. On the other hand, hypoxic stress in cancer stimulates the inositol requiring enzyme 1α (IRE1)/XBP1 arm of the UPR to blunt immune elimination of cancer cells, by decreasing the expression of major histocompatibility complex 1 (MHC1) molecules in antigen-presenting dendritic cells, thereby weakening the function of CD8^+^ lymphocytes to kill cancer cells [45]. Another aspect of MMP11 role in mediating cancer progression is the altered mitochondrial unfolded protein response (UPR^mt^) observed in tumors from PyMT^Tg^; MMP11^Tg^ mice as demonstrated by the decreased expression of important proteins of the UPR^mt^ such as the chaperone proteins HSP10 and HSP60 or the mitochondrial protease CLPP, which role is to eliminate misfolded or unfolded proteins in the mitochondrial matrix. This axis of the UPR^mt^ is referred as the CHOP pathway [46]. These results are surprising, in the sense that tumor aggressiveness is often associated with mitochondria fitness and adaptation to stress. One possibility is that other axes of the UPR^mt^ may be activated. We measured the transcripts of proteasome components and *Sirt3*, the 2 other branches of the UPR^mt^ [31]. We found a decrease in *Pmsb1* and *Pmsd1* expression in PyMT^Tg^; MMP11^Tg^ and an increase of their expression in PyMT^Tg^; MMP11^KO^ as compared to their respective controls; however, *Sirt3* expression was significantly increased in PyMT^Tg^; MMP11^Tg^ mice as compared to controls and decreased *Sirt3* in PyMT^Tg^; MMP11^KO^ tumors. Interestingly, the SIRT3 axis of UPR^mt^ was shown to induce the antioxidant proteins superoxide dismutase 2 (SOD2) and catalase, and the subsequent elimination of irreversibly damaged mitochondria through mitophagy [47]. The SIRT3 arm molecular signature of the UPR^mt^ was recently shown to contribute to breast cancer invasiveness and may be an essential mechanism for cancer cells to adapt to proteotoxic and mitochondrial stress, a process called mitohormesis [48].

Another observation that we present is that the presence of MMP11 is associated with a decrease in the expression of the proteasome subunits Psmb1 and Psmd1 and may result in decreased proteasome activity. Mitochondrial dysfunction produced by impaired ETC and ROS generation was shown to inhibit proteasome activity [33,34] because of ATP depletion, ATP being crucial for proteasome assembly. Moreover, we observed a significant increase in AMPK phosphorylation, a kinase induced upon nutrient deprivation. It is tempting to speculate that non-degraded stressed mitochondria may amplify the proteotoxic and mitochondrial stress contributing further to ATP-depletion and increase in AMPK activation, a known activator of autophagy [49]. To give weight to this hypothesis, a recent report has shown that accumulation of ubiquitin-protein conjugates was correlated with decrease in cellular bioenergetics and increase in AMPK activation and autophagy [50]. Autophagy is the process by which the cell recycles the constituents of irreversibly damaged organelles to generate energy and recover precursors necessary for cell growth. This hypothesis and the occurrence of autophagy need to be verified in our model.

Finally, metabolomics analyses of tumor samples from our two mouse models (GOF and LOF) reveal a significant change in metabolite profile as compared to their respective controls. Hence, the increase in lactate production and glutamine metabolism are associated with increased tumorigenesis and tumor aggressiveness [21]. Lactate can be used as an extra nutrient to fuel cancer cells with a supplementary source of energy and glutamate formed from glutamine can be transformed to α-ketoglutarate that will feed the tricarboxylic cycle to generate energy in the mitochondria. Moreover, besides activation of lactate and glutamine metabolism in the tumors of PyMT^Tg^; MMP11^Tg^ mice, we observe an increase in serine metabolism as shown by the predicted increase in glycine levels. This metabolism is a diverted branch of aerobic glycolysis, known as the phosphoglycerate dehydrogenase (PHGDH) pathway, which aims to promote nucleotide synthesis. Interestingly, PHGDH overexpression is associated with certain breast subtypes, and reduction of PHGDH reduced breast tumors growth, while ectopic PHGDH expression in mammary epithelial cells induced phenotypic alterations that may predispose cells to transformation [37,38]. Thus, PHGDH could be a metabolic target of MMP11 in tumors. Finally, elevation of the non-essential amino acid alanine observed in PyMT^Tg^; MMP11^Tg^ tumors has been recently associated with increased breast tumorigenesis through alanine aminotransferase (ALAT)-mediated transformation of pyruvate to α-ketoglutarate, which is important to shape metastatic niche in breast cancer [51]. Altogether, MMP11 stands as an important metabolic regulator both in physiological energy homeostasis and in the context of breast cancer (BC). In the PyMT model, MMP11 influences tumor growth and metabolism, whether its catalytic activity is required for these functions remains uncertain. The protein is predominantly present under its catalytically inactive pro-form in the skin of the GOF model. Therefore, one can hypothesize that MMP11 acts independently from its catalytic activity, for example, as a signaling molecule or by interfering with the protease/inhibitor network. Nevertheless, pharmacological targeting of this protein using either antisense oligonucleotides, CRISP-Cas9 technology or monoclonal antibodies directed against MMP11 may prove efficient to impede rapid cancer progression by acting on multiple biological processes such as proliferation, apoptosis, metabolism, and organelle unfolded protein responses.

## 4. Material and Methods

### 4.1. Generation of Mice Cohorts

This study was approved by the Ethical Committee (IGBMC and ICS ComEth, 2014-0039 and 2014-0122). In the GOF model, MMTV-PyMT^Tg^ male mice (FVB/N-Tg(MMTV-PyVT)634Mul/J) were obtained from The Jackson Laboratory and crossed with K14-MMP11^Tg^ female mice (FVB/N background). The first mice generation were genotyped. In the LOF model, MMTV-PyMT^Tg^ male mice (FVB/N background) were crossed with MMP11^KO^ female mice (129/SvJ background). The first mice generation was genotyped. Then, PyMT^Tg^; MMP11 −/+ male mice were crossed with PyMT^WT^; MMP11 −/+ females and their offspring mice generation were genotyped. Age-matched PyMT^Tg^; MMP11^WT^ and PyMT^Tg^; MMP11^Tg^ female mice, on one hand, and age-matched PyMT^Tg^; MMP11^WT^ and PyMT^Tg^; MMP11^KO^ female mice were randomly divided and used for experiments. Each group in different experimental cohorts contains 6–8 mice.

### 4.2. Mice Genotyping

The genotyping of the PyMT transgene was performed by standard PCR as recommended by the Jackson Laboratory. In brief, PCR reaction from mouse tail DNA was carried out as follows: 94 °C for 30 s, 64 °C for 1 min, 72 °C for 1 min, 35 cycles. Likewise, K14-MMP11 genotyping was done by standard PCR reaction using tail DNA: 94 °C for 1 min, 60 °C for 20 s, 72 °C for 1 min, 33 cycles; MMP11 KO/WT gene PCR reaction: 94 °C for 15 s, 62 °C for 15 s, 72 °C for 1 min, 33 cycles. Primers used for genotyping are listed in Table S1.

### 4.3. Mice Weight and Tumor Measurement

Mice weight was measured by electronic balance twice a week. Caliper was used to measure the tumor length, width, and height twice a week. Tumor volume was calculated following the formula (4/3) × 3.14159 × (length/2) × (width/2) × (height/2).

### 4.4. Carmine-Alum Red Staining

The #4 mammary glands were spread onto glass slides and fixed in Carnoy’s solution (75% glacial acetic acid, 25% absolute EtOH) overnight. A sequential rehydratation in 2 steps (100% EtOH and 70% EtOH) of one hour each was followed by 30 min in distilled water. Then, carmine-alum (Sigma-Aldrich, C1022, St-Quentin Fallavier, France) staining solution was added overnight. Dehydratation was done in a stepwise incubation in 70% EtOH, 95% EtOH, and 100% EtOH for 1 h each step. The slides were fixed in Histosol overnight and mounted with Permount.

### 4.5. HE Staining

Paraffin-embedded #4 mammary gland tumor tissue slides were immersed into 100% Histosol for 2 times, 5 min each. Then, slides were sequentially washed in 100%, 90%, 80% ethanol and H_2_O for 5 min each before being placed in Harris Hematoxylin staining solution for 3 min. Slides were washed in acid alcohol for 2 s, in running tap water for 3 min. Next, they were stained for 30 s in 0.1% aqueous eosin Y, rinsed in tap water for 30 s, dehydrated in 80%, 90%, 100% ethanol for 5 min each, fixed by two washes of 100% histosol, 5 min each and mounted with Permount.

### 4.6. TUNEL Assay

For this assay, we used the TUNEL kit from Abcam company (#ab206386, 75010 Paris, France). Paraffin-embedded tumor tissue slides were treated as recommended by the user guide booklet. Slides were mounted using Histosol mounting media.

### 4.7. Immunofluorescent Staining

Paraffin-embedded #4 mammary gland tumor tissue slides were immersed into 100% Histosol for 2 times, 5 min each, followed by 100%, 90%, 80% ethanol and H_2_O for 5 min each. Tissue specimens were rinsed 1 time in phosphate-buffered saline (PBS) 1×. Antigen unmasking was performed by incubation at 95 °C in Tris-EDTA buffer for 20 min. Permeabilization was done in PBS 1× containing 0.1% Triton X-100 for 1 h. Blocking in bovine serum albumin (BSA) 5% for 1 h. The primary antibody (Rabbit anti-Ki67, Bethyl IHC-00375, 1:500; Rabbit anti-p-eIF2α, #3597s, 1:500, CST) was incubated in BSA 5% at 4 °C for overnight. The slides were rinsed 3 times in PBS 1×, 5 min each and incubated with secondary antibodies in PBS 1× for 2 h. Nuclei were stained with Hoechst dye diluted in PBS 1× for 10 min, washed with PBS 1× for 2 times, 5 min each. Coverslip was mounted with Prolong Gold antifade. Images were taken using an inverted laser confocal fluorescence microscopy. Primary and secondary antibodies for immunofluorescence staining are listed in Table S2 in the supporting information file attached to the manuscript.

### 4.8. Western Blot

Mice tumor tissues were taken from #1 mammary gland and protein extracts were obtained by tissue grinding in RIPA lysis buffer. Protein concentrations were quantified by the BiCinchoninic acid Assay (BCA) method. Protein extracts were separated on a 12% SDS-PAGE gel and transferred onto nitrocellulose membranes. Membranes were incubated with primary antibodies (Rabbit anti-Bcl2, 1:500, Abcam, ab59348; Rabbit anti-IGFBP1, 1:1000, Abcam, ab181141; Rabbit anti-GAPDH, 1: 5000, Sigma-Aldrich, G9545; Rabbit anti-pAKT, 1:1000, Cell Signaling Technology (CST), #4060; Rabbit anti-AKT, 1:1000, CST, #9272; Rabbit anti-pFoxO1, 1:1000, CST, #9461; Rabbit anti-FoxO1, 1:1000, CST, #2880; Rabbit anti-pAMPK, 1:1000, CST #2535; Rabbit anti-pAMPK, 1:1000, CST #2532; Rabbit monoclonal anti-MMP11, IGBMC#3143). Primary and secondary antibodies as well as the quantification method are detailed in the supporting information file attached to the manuscript (Table S3). Uncropped western blots are shown in supplementary Figures S3–S5.

### 4.9. RT-qPCR

Frozen mice tumor tissues from #1 mammary gland were homogenized in Trizol^®^ reagent (Sigma-Aldrich, T9424, St-Quentin Fallavier, France) and total RNA were extracted and reverse transcribed using SuperScript^®^ IV Reverse transcriptase kit (Invitrogen by Life technologies, kit #18090050, Paisley, UK) following the manufacturer’s recommendations. Normalized gene expression levels were quantified by real-time quantitative RT-PCR using SYBR^®^ Green JumpStart™ Taq ReadyMix™ (kit #S4438, Sigma-Aldrich, St-Quentin Fallavier, France) and specific primers listed in Table S4.

### 4.10. HRMAS NMR Metabolomics Analysis

For all PyMT^Tg^; MMP11^Tg^ and PyMT^Tg^; MMP11^KO^ mice and their respective PyMT^Tg^; MMP11^WT^ controls, the tumors were extracted, snap frozen, and stored at −80 °C until analysis. Tumor samples (mass between 12 and 20 mg) were prepared at −20 °C and introduced into a disposable 25 µL KelF insert. HRMAS NMR spectra were acquired on a Bruker (Karlsruhe, Germany) Avance III 500 spectrometer operating at a proton frequency of 500.13 MHz and equipped with a 4-mm triple-resonance gradient HRMAS probe (^1^H, ^13^C, and ^31^P). The temperature was maintained at 4 °C throughout the acquisition time in order to reduce the effects of tissue degradation during the spectrum acquisition. A one-dimensional (1D) proton spectrum using a Carr-Purcell-Meiboom-Gill (CPMG) pulse sequence was acquired with a 285-µs inter-pulse delay and a 10-min acquisition time for each tissue sample. The number of loops was set at 328, giving the CPMG pulse train a total length of 93 ms. Spectra were normalized according to each sample weight and calibrated using the internal intensity of a reference solution containing a known amount of lactate. The peaks of interest were integrated using a routine developed under MATLAB (MATLAB 7.0; Mathworks, Natik, MA, USA). Quantification results were expressed as nmol/mg of tissue.

The algorithm to determine the expected metabolite level alterations (ADEMA) network analyses using mutual information was applied to the metabolite quantification values [36]. ADEMA includes information on the metabolic pathway in a unidirectional or bidirectional manner. The network was constructed using Salway’s work [52]. Using the metabolic network topology, the ADEMA algorithm evaluates the change in groups of metabolites between concentration data from two experimental groups instead of analyzing metabolite concentrations one by one. Based on mutual information, the algorithm determines whether some metabolites are biomarkers when considered together, and it can predict the direction of the expected change per metabolite depending on the metabolic network topology considered.

### 4.11. Data Analysis

The quantification of hyperplasia and neoplastic lesions area, tumor necrosis area, TUNEL staining cells, Ki-67 staining cells, *p*-eIF2α relative intensity, and Western blot were performed by Image J 1.51n. Statistical analysis was performed by Graphpad Prism 6.

## 5. Conclusions

In conclusion, we report that MMP11 inhibits cell death and promotes tumor growth by activating the IGF1/AKT/FoxO1 pathway in the MMTV-PyMT mouse model of mammary gland tumor. Expression of MMP11 is regulating important metabolic pathways such as the metabolic reprogramming from oxidative phosphorylation to aerobic glycolysis, and adaptive organelle processes following proteotoxic stress such as the UPR^ER^ and UPR^mt^. These pathways enable cancer cells to cope with nutrient availability, allowing them to survive in harsh conditions such as the presence of ROS.

## Supplementary Materials

The following are available online at https://www.mdpi.com/2072-6694/12/9/2357/s1, Figure S1: Generation of PyMT^Tg^; Mmp11^Tg^ and PyMT^Tg^; Mmp11^KO^ animals and their respective controls., Tumor development in MG #4 and MG #5 and MMP11 expression in the skin of Mmp11^Tg^, Figure S2: MMP11 expression in the skin of MMP11Tg and tumor development in MG #4 and MG #5, Figure S3: Uncropped blots from Figure 2C, Figure S4: Uncropped blots from Figure 3B, Figure S5: Uncropped blots from Figure 5D, Table S1: List of primers used to amplify genomic DNA extracted from tails of PyMT^Tg^; Mmp11^Tg^ and PyMT^Tg^; Mmp11^KO^ animals and their control littermates, Table S2: List of antibodies used for immunofluorescence on paraffin-embedded tumor tissue, Table S3: List of antibodies used for immunoblots, Table S4: List of primers used for qRT-PCR quantification of gene expression.
