# Supplementary material for: Matrix Metalloproteinase-11 Promotes Early Mouse Mammary Gland Tumor Growth through Metabolic Reprogramming and Increased IGF1/AKT/FoxO1 Signaling Pathway, Enhanced ER Stress and Alteration in Mitochondrial UPR
Source: Cancers (Basel). 2020 Aug 20;12(9):2357. doi: 10.3390/cancers12092357 (PMC7565046; doi:10.3390/cancers12092357)
Supplement: Supplementary file 1 [file cancers-12-02357-s001.pdf]

Supplementary Materials:

Matrix Metalloproteinase-11 Promotes Early Mouse Mammary Gland Tumor Growth through Metabolic Reprogramming and Increased IGF1/AKT/FoxO1 Signaling Pathway, Enhanced ER Stress and Alteration in Mitochondrial UPR

Table S1. List of primers used to amplify genomic DNA extracted from tails of $PyMT^{Tg}$; $MMP11^{Tg}$ and $PyMT^{Tg}$; $MMP11^{KO}$ animals and their control littermates.

	Forward	Reverse
PyMT	GGAAGCAAGTACTTCACAAGGG	GGAAAGTCACTAGGAGCAGGG
$MMP11^{Tg}$	CGGTTTCCACCATCCGAGGA	GTGGAAACGCCAATAGTCTCC
$MMP11^{KO}$	GTGGAAACGCCAATAGTCTCC	GCCGCTTTTCTGGATTCATCG
$MMP11^{WT}$	GTGGAAACGCCAATAGTCTCC	TTCTAACATCCCTCTGGGCTC

Table S2. List of antibodies used for immunofluorescence on paraffin-embedded tumor tissue.

**Primary Antibody**			
Rabbit anti-Ki67	Bethyl IHC-00375	1:500	TBS 1X + 5% BSA
Rabbit anti-peIF2α	CST #3597s	1:500	TBS 1x + 3% BSA
Hoescht	Sigma-Aldrich 33258	1:400	TBS 1X
**Secondary Antibody**			
AlexaFluor 488 donkey anti-rabbit IgG	Thermo Fisher Scientific A21206	1:1000	TBS 1X

Table S3. List of antibodies used for immunoblots.

Primary Antibodies	Reference	dilution	
Rabbit anti-Bcl2	Abcam ab59348	1:500	TBS 1X-Tween 0.1% + 5% BSA
Rabbit anti-IGFBP1	Abcam ab181141	1:1000	
Rabbit anti-p-AKT	CST #4060	1:1000	
Rabbit anti-AKT	CST #9272	1:1000	
Rabbit anti-pFoxO1	CST #9461	1:1000	
Rabbit anti-FoxO1	CST #2880	1:1000	
Rabbit p-AMPK	CST #2535	1:1000	
Rabbit AMPK	CST #2532	1:1000	
Rabbit anti-MMP11	IGBMC N°3142 and 3143	1:500	TBS 1X-Tween 0.1% + 3% milk
Rabbit anti-GAPDH	Sigma G9545	1:5000	TBS 1X-Tween 0.1% + 5%BSA
**Secondary Antibodies**			
Peroxidase-conjugated Affinipure Goat Anti-Rabbit IgG		1:10000	TBST 1X

Western Blot, Detection Method and Quantification

Mice tumor tissues were taken from #1 mammary gland and protein extracts were obtained by tissue grinding in RIPA lysis buffer. Protein concentrations were quantified by the BCA method. Protein extracts were separated on a 12% SDS-PAGE gel and transferred onto nitrocellulose membranes. Membranes were blocked with 5% nonfat dry milk in TBS 1X-Tween 0.1% and then

incubated with primary antibodies followed by secondary antibody. GAPDH was used as a loading control.

For MMP11 detection in the skin, about 200 mg of tissue sample was frozen in liquid nitrogen and directly ground in 2X Laemmli buffer. Samples were sonicated and centrifuged to eliminate debris. Protein concentration was estimated by western blot analysis and Ponceau S staining. Conditioned media containing active MMP11 was obtained by transfection of PQCXIP-hMMP11 plasmid in HeLa cells and incubating cells in serum-free medium containing 0.05% BSA for 48 hours.

To generate the anti-MMP11 rabbit polyclonal antibodies, a peptide was synthetized (PK78: FYTFRYPLSLSPDDC). Its sequence corresponds to residues 240-253 and to 236-249 for the mouse and human MMP11 protein sequences, respectively. Human and mouse protein sequences are identical in this region. The PK78 peptide was coupled to ovalbumin through an additional cysteine residue at the carboxy-terminal end and injected into two New Zealand rabbits. Two immunoreactive sera (# 3142 and # 3143) were obtained. To gain into specificity, both sera were affinity-purified against the synthetic peptide coupled to Sulfolink coupling gel (Pierce, Rockford, IL) using conditions described by the manufacturer.

Membranes were revealed with the Amersham™ Imager 600 (GE Healthcare, Life sciences) using Amersham™ ECL™ Start Western Blotting Detection Reagent (GE Healthcare, Life Sciences, RPN3244).

The quantification of western blot images is performed by Image J 1.51n. Western blot images (.tif file) are converted to 16-bit format. Each band (and background) is circumscribed with rectangular ROIs of the same size, and signal intensity is measured in each ROI. Background values are subtracted to other values. For each specific protein, quantifications were performed on 2 independent western blot images.

Table S4. List of primers used for qRT-PCR quantification of gene expression.

	**Forward**	**Reverse**
Cd36	GATGTGGAACCCATAACTGGATTCAC	GGTCCCAGTCTCATTTAGCCACAGTA
Pparα	AGGAAGCCGTTCTGTGACAT	TTGAAGGAGCTTTGGGAAGA
Aco	CCCAACTGTGACTTCCATT	GGCATGTAACCCGTAGCACT
Acc1	GACAGACTGATCGCAGAGAAAG	TGGAGAGCCCCACACACA
Acc2	CCCAGCCGAGTTTGTCACT	GGCGATGAGCACCTTCTCTA
Ndufb5	CTTCGAACTTCCTGCTCCTT	GGCCCTGAAAAGAACTACG
Sdha	GGAACACTCCAAAAACAGACCT	CCACCACTGGGTATTGAGTAGAA
Sdhc	GCTGCGTTCTTGCTGAGACA	ATCTCCTCCTTAGCTGTGGTT
Cox2	AATTAGCTCCTTAGTCCTCT	CTTGGTCGGTTTGATGTTAC
Cox5b	AAGTGCATCTGCTTGTCTCG	GTCTTCCTTGGTGCCTGAAG
Atp5b	GGTTCATCCTGCCAGAGACTA	AATCCCTCATCGAACTGGACG
Hsp10	CTGACAGGTTCAATCTCTCCAC	AGGTGGCATTATGCTTCCAG
Hsp60	ACAGTCCTTCGCCAGATGAGAC	TGGATTAGCCCCTTTGCTGA
Clpp	CACACCAAGCAGAGCCTACA	TCCAAGATGCCAAACTCTTG
Phb	TCGGGAAGGAGTTCACAGAG	CAGCCTTTTCCACCACAAAT
Phb2	CAAGGACTTCAGCCTCATCC	GCCACTTGCTTGGCTTCTAC
Mct1	GCATTTCCCAAATCCATCAC	CGGCTGCCGTATTTATTCAC
Mct4	GGTCAGCGTCTTTTTCAAGG	CCGTGGTGAGGTAGATCTGG
Ldha	AGACAAACTCAAGGGCGAGA	CAGCTTGCAGTGTGGACTGT
Ldhb	TAAGCACCGTGTGATTGGAA	AGACTCCTGCCACATTCACC
Xbp1	GGTCTGCTGAGTCCGCAGCAGG	AGGCTTGGTGTATACATGG
Atf4	CCTTCGACCAGTCGGGTTTG	CTGTCCCGGAAAAGGCATCC
Atf6	CTGTGCTGAGGAGACAGCAG	CTTGGGACTTTGAGCCTCTG
Psma1	TGCGTGCGTTTTTGATTTTAGAC	CCCTCAGGGCAGGATTCATC
Psmb1	CGTTGAAGGCATAAGGCGAAAA	TTCCACTGCTGCTTACCGAG
Psmd1	GTGATAAAACACTTTCGAGGCCA	TGAATGCAGTCGTGAATGACTT
Sirt3	ACAGCTACATGCACGGTCTG	GGGAGGTCCCAAGAATGAGT
Mmp11	ATGTACTGAATGCCCGGAAC	TCGTGCACCTCAGTGAAAGT
Gapdh	ACTGGCATGGCCTTCCGTGTTC	TCTTGCTCAGTGTCCTTGCTGG
36b4	AGATTCGGGATATGCTGTTGG	AAAGCCTGGAAGAAGGAGGTC

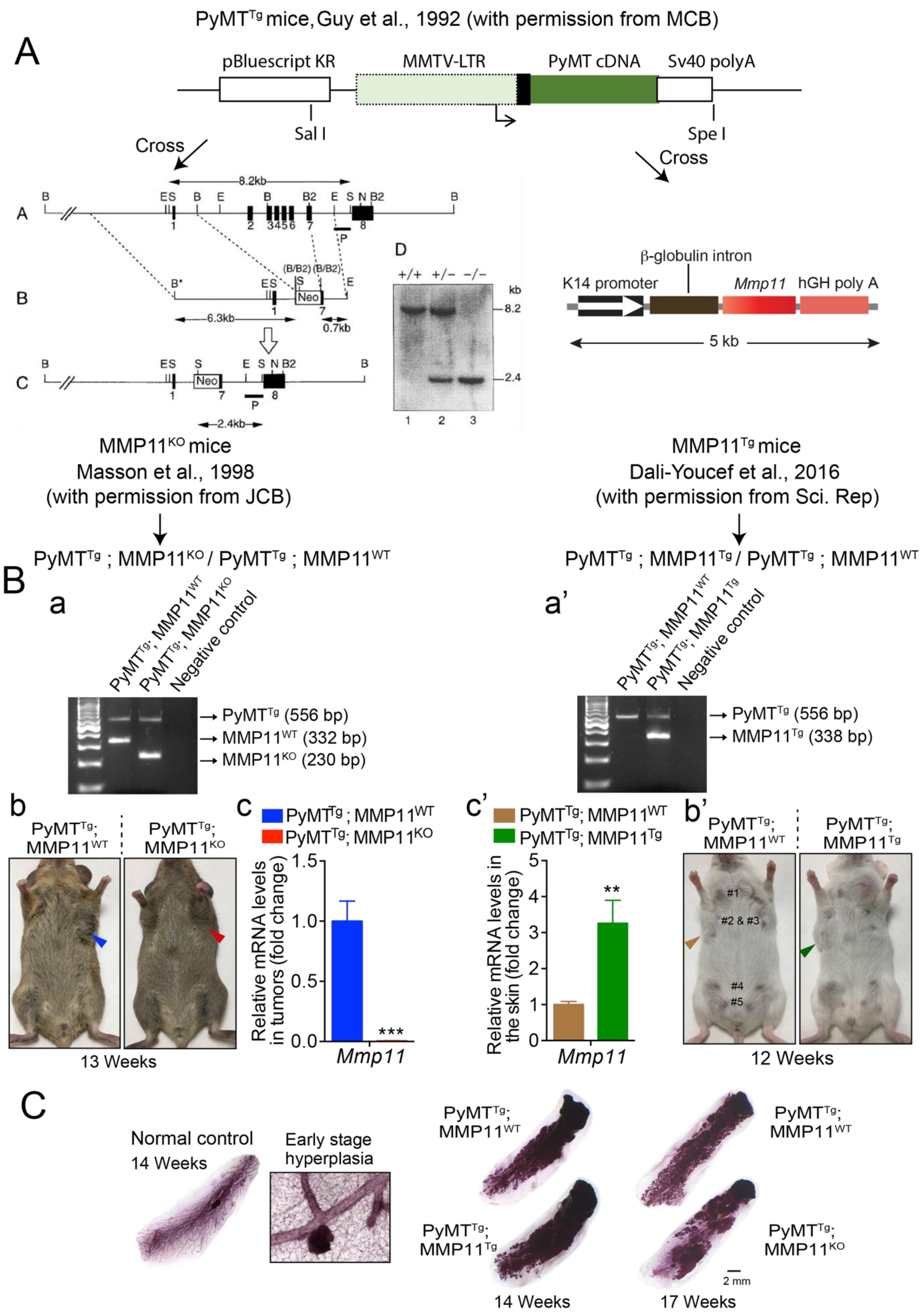

Figure S1. Generation of PyMT^{Tg}; MMP11^{Tg} and PyMT^{Tg}; MMP11^{KO} animals and their respective controls. **A.** Constructs used to generate double transgenic animals from PyMT^{Tg} mice and either gain- or loss-of-function MMP11^{Tg} and MMP11^{KO} mice. Single transgenic models have already been published

as mentioned in the figure; **B.** Identification of $PyMT^{Tg}$ and $MMP11^{KO}$ double transgenic genotypes **(a)** and $PyMT^{Tg}$ and $MMP11^{Tg}$ genotypes **(a′)** from genomic DNA obtained from the tails. **(b)** Representative mice displaying mammary gland tumors from $PyMT^{Tg}$; $MMP11^{KO}$ mice and their controls; **(b′)** Representative mice displaying mammary gland tumors from $PyMT^{Tg}$; $MMP11^{Tg}$ mice and their controls and the mammary glands identification number are shown; **(c)** *Mmp11* gene expression levels in tumors from $PyMT^{Tg}$; $MMP11^{KO}$ mice and their controls. As expected, *Mmp11* expression is barely detected in tumors from $PyMT^{Tg}$; $MMP11^{KO}$ mice; **(c′)** *Mmp11* expression in the skin from $PyMT^{Tg}$; $MMP11^{Tg}$ mice and their controls; **C.** Carmine-alum red staining of mammary glands from $PyMT^{Tg}$; $MMP11^{Tg}$ and $PyMT^{Tg}$; $MMP11^{KO}$ mice and their controls. Early stage hyperplasia in control mice is shown as well as tumors from double transgenic mice and their respective controls.

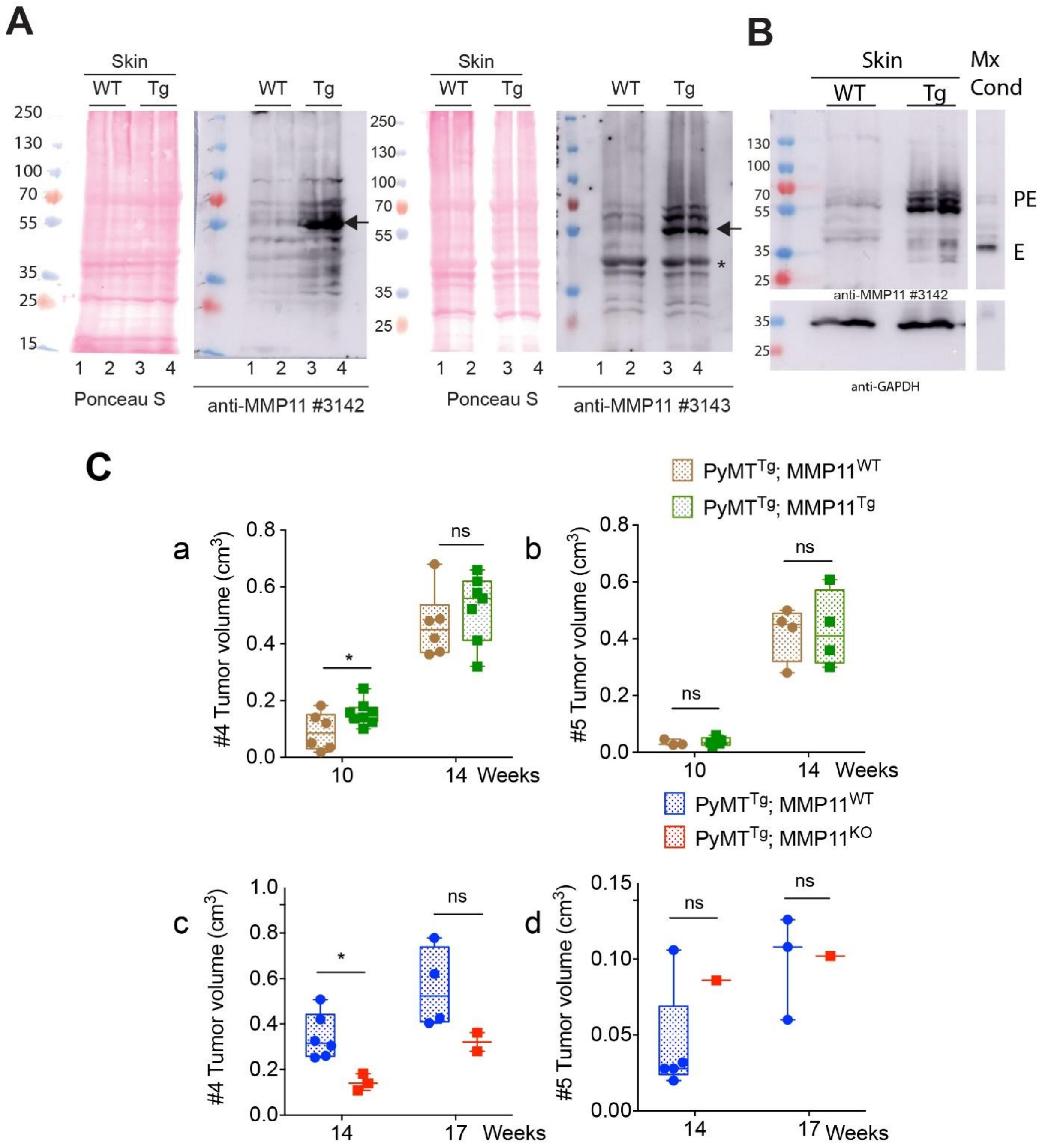

Figure S2. MMP11 expression in the skin of MMP11^{Tg} and tumor development in MG #4 and MG #5. (**A**) MMP11 expression in the skin of MMP11^{Tg} (Tg) and control (WT) mice (n = 2). Two polyclonal sera against MMP11 were utilized: #3142 and # 3143; on duplicate blots. On the left, total transferred proteins were visualized using Ponceau S staining, on the right, western blot analysis. Arrows show the presence of MMP11 at the size of the inactive pro-enzyme (55 kDa); (**B**) Analysis of the MMP11 molecular forms present in the skin of MMP11^{Tg}. Skin samples (~20µg) from control and Tg mice (n = 2) were loaded and analyzed using anti-MMP11 and anti-GAPDH rabbit antibodies. Conditioned medium (Mx Cond) of HeLa cells transfected with a MMP11 expression vector was loaded to show the size of the secreted and active form (45 KDa). Note that in the skin of transgenic mice the pro-form (PE) is predominantly expressed, only a small amount the active form (E) is detected. *: non-specific signal**;** **(C)** Tumor volume in MG # 4 and # 5 double transgenic animals from PyMT^{Tg} mice and either gain- or loss-of-function MMP11^{Tg} and MMP11^{KO} mice as in Figure 1.

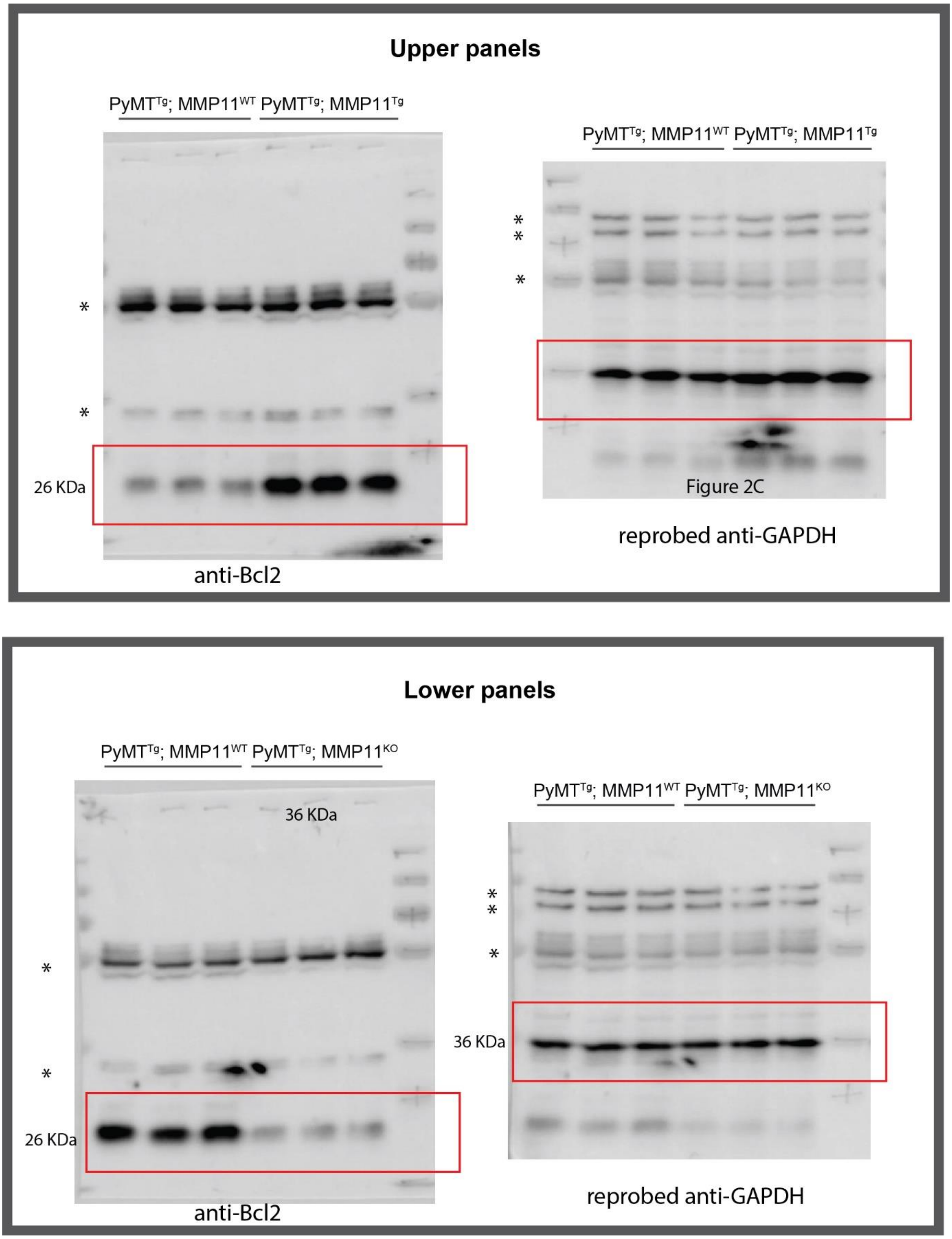

Figure S3. Uncropped blots from Figure 2C.

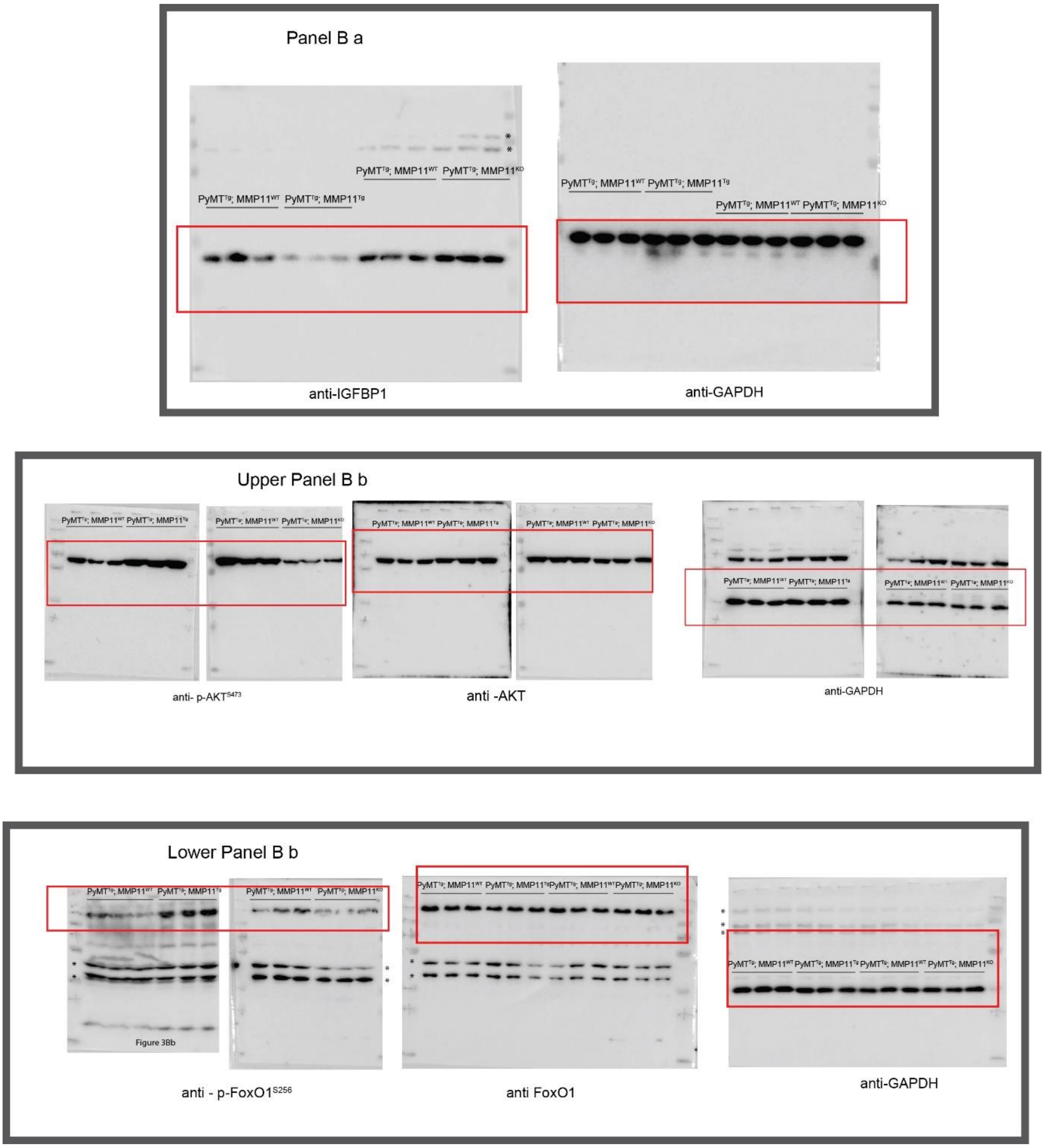

Figure S4. Uncropped blots from Figure 3B.

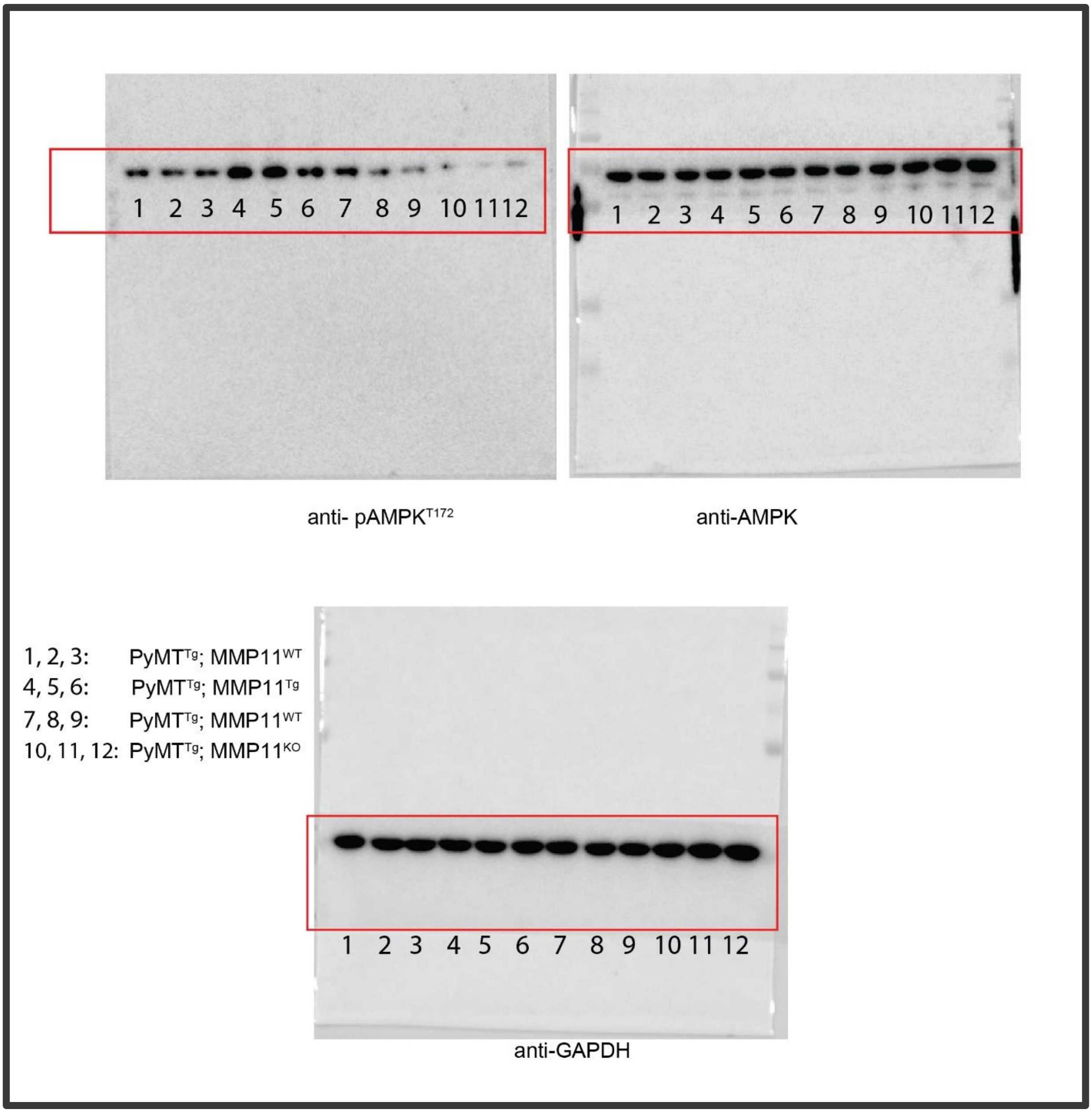

Figure S5. Uncropped blots from Figure 5D.
